# Bibliographical

**Published:** 1874-04

**Authors:** 


### BIBLIOGRAPHICAL.

A system of Midwifery, including the diseases of pregnancy and the puerperal state. By William Leishman, M.D. Regius Professor of Midwifery in the University of Glascow, etc., etc. With one hundred and eighty-two illustrations. Philadelphia: Henry C. Lea, 1873.

The author introduces himself to the reader by giving a history of midwifery and some practical remarks upon the anatomy of the pelvis, etc. He notices parturition in the primates in the various races, and gives as a reason for the main cause of comparative difficulty of parturition in the human species, the erect posture. His definition of the word "midwifery," as used in his work, is employed in a more extended sense than by Rigby and other English authors, and by him is made to signify "that science and art which has for its object, the management of women and her offspring during pregnancy, labor, and the puerperal state."

Further on in his work the pelvis; the female organs of generation; the graafian vesicles and their development; the ovum; the phenomena of ovulation, etc., are considered. He devotes a chapter to menstruation and conception. He regards the discharge as a hemorrhage and not a secretion as contended by Churchill and others.

The arrest of coagulation—upon which, in part, was predicated the idea of the discharge being secretory in character—he attributes the fact of mixture of the menstrual blood with the acid secretions of the vagina. He regards the true source of this "hemorrhage," "the mucous membrane which lines the cavity of the uterus" and that ovulation and menstruation "are mutually dependent on each other." He rejects the theory of M. Ponchet, in which the mucous membrane is supposed to be deciduous, not only at the termination of pregnancy but at each menstrual period. He gives his adhesion rather to the views of Kolliker and others, that the mucous membrane becomes thickened and that a certain change takes place in the epithelium during menstruation. He very fully discloses the observations and researches of various authors in regard to the development of the ovum and the development of the embryo and fœtus.

Ample space and attention is given to pregnancy, and the signs of pregnancy, the duration of pregnancy and super-fœtation. He speaks of plural pregnancy and extra-uterine pregnancy at considerable length. The symptoms of the latter he declares are "far from being definite and distinct." The "uterine effort at the end of the ninth month is, he avers "a physiological fact of surpassing interest." He is convinced that these uterine pains originate neither in the fœtus or the uterus, but probably in the ovary and may "generally be looked for at the tenth menstrual period of impregnation." He devotes a chapter to abnormal development and two to the diseases of pregnancy. The latter are clearly and forceable given, and the treatment of these various disorders, which must occupy largely the attention of the practitioner, is as practical as the most practical could wish. "Morning sickness" he notices at length and believes there are no class of remedies attended with such beneficial results as effervescing draughts." He prefers the citrate of magnesia, and refers, approvingly to the "portion de Riviere." He discusses the question of inducing the premature expulsion of the fœtus, when all remedies fail and the life of the patient is placed in jeopardy. No proposition can be clearer. The duty of the practitioner—from the American stand-point—is to "go forward" promptly in such cases. In puerperal albuminuria he recommends the avoidance of all antiphlogistic treatment, a condition, he thinks calling more for a tonic treatment and a generous diet. Where, however, the general system seems to participate in the morbid process—with lumbar pain and febrile excitement—he recommends the application of a few leeches to the loins, followed by diligent fomentation—with baths of various kinds. He does not esteem diuretics, for the most part, as valuable or serviceable.

He devotes a chapter to "Labor and its Phenomena," and another to the "Management of Natural Labor." The instructions given in the latter are clear, concise, and withal fully up to the times—adapted especially to the wants of the practitioner who desires rather to "act" than to revel in theories. "After the termination of the first stage," he remarks: "It not unfrequently happens that the anterior lip of the os remains in an œdematous condition, indicative of pressure of the anterior uterine wall between the presenting part and the symphysis pubis." This condition becoming an impediment to the progress of the labor, shall the accoucheur interfere? The maxim of "meddlesome midwifery is dangerous" in this as other conditions which seem to call for "meddlesome midwifery," stares him in the face. In the condition given, Rigby and others advise, "Hands off." Our author, with whom we fully agree, demurs. He truly says, as we can testify, that if the accoucheur will place his finger upon the part and gently, at each pain, attempt to carry it over the head, "it will expedite the labor in a majority of cases." In cases where the rigidity of the os is so great as to retard the labor, he prefers small doses of tarterized antimony to the old practice of blood-letting. Chloroform he esteems highly for the purpose of relaxing the rigid os. In all edious labors, he recommends the use of the stethoscope to ascertain the vitality and vigor of the fœtus. He condemns "support" to the perineum as "irrational and useless in all cases, and undoubtedly hurtful in others." He asserts that "the practitioner who never puts his hand to the perineum will have fewer cases of ruptured perineum than he who admits support in any form as applicable to every case of labor." In this he agrees with Prof. Goodell, of Philadelphia. He gives the "mechanism of labor" ample attention, devoting two chapters to the subject. These contain some objectionable features to American-taught obstetricians. It is worthy of remark, and generally conceded across the Atlantic, that in the department of Obstetrics and Gynæcology, American authors take rank second to those of no other portion of the world: and the teachings of Hodge and other Americans we regard as superior, in many respects, to those of any foreign obstetrical author. In the practical details, and especially in reference to the practical instructions given in the various disorders attending pregnancy, the puerperal state, etc., English writers—among whom our author stands preëminent—are unequalled. To return: Our author devotes a chapter to pelvic presentations and another to transverse presentations and complicated presentations. The "premature expulsion of the ovum" is noticed at length, but is handled with no unusual merit or originality. Considerable space is given to the discussion of hemorrhage before and after delivery. Other chapters follow, of which our space forbids an extended notice. Inversion of the uterus; rupture of the uterus and deformities of the pelvis; and the forceps. Upon the latter subject we prefer following Hodge, both as to instruments and their application.

The succeeding chapters are devoted to the vectis; fillet; blunt hooks; turning; embryotomy; histerotomy and allied operations; induction of premature labor; labor obstructed by maternal soft-parts; obstruction depending on the state of the ovaries; uterine inertia and precipitate labor; the puerperal state; the newly born child; phlegmasia dolens; puerperal insanity; puerperal eclampsia; puerperal fever; pelvic peritonitis, etc., forming a volume of very great value to the practitioner. We have scanned the work hurriedly. We have noted, however, few faults, and find much to commend. It is a volume no practitioner should fail to read. It is fully abreast with the age, and rich in practical instruction.

W. T. G.

**Specific Medication and Specific Medicines. Revised, with an Appendix, etc. By John M. Scudder, M.D., Prof. of the Principles and Practice of Medicine in the Eclectic Medical Institute, etc. Fifth edition. Cincinnati: Wilstach, Baldwin & Co., printers. 1874.**

Since the earliest dawn of medical science, the professional mind has delighted itself in seeking for and dwelling upon "specific medication," "specific medicines" and "panaceas for every woe." And yet, the voice of sadness echoing down the steep of centuries, repeats the story, like Poe's sepulchral raven—never more! Specifis,—yes, *specifies*, haunt the imagination of many even in this blessed era of science and rigid analysis of physical, physiological and pathological phenomena. The human mind, unless kept under constant restraint, will follow a phantasmagoria of its own creation upon any subject—the most important—even when the future destiny of the soul itself is at stake.

The work before us is, we believe, an honest effort to simplify, the application of a comparatively small number of remedial agents, and an earnest hunt for specific medication by an American Eclectic, it is true, but by one who, like Saul, towers head and shoulders above the disciples of that school, in his investigations of our indigenous materia medica.

Dr. Scudder, in spite of his exclusive dogmas and his searchings for specific medicines, is a physician. Like all intelligent medical men, of whatever bias of mind, he sees clearly the absurdity of the doctrine of "universal specifics;" hence he qualifies his "specific medication" by basing it upon "specific diagnosis;" *i. e.*, if an intelligent physician can, with certainty, locate the pathologica trouble, he can specifically, by medication, remove that trouble] This, it will be seen, is a very necessary qualification, but one which, nevertheless, borders closely upon the realm of mysticism.

Our author, in his honest efforts to attain to greater certainty in his therapia—confined largely to indigenous agents—has become an earnest worker, and, we trust, an accurate expounder in his chosen field of investigation. He, with his so-called Eclectic colaborers, have made—leaving out of view the exclusiveness of his school—valuable additions to the general stock of remedial agents; which, in truth, is the beginning and the end of American Eclecticism.

The work, as intimated, bears the impress of a sincere and laborious thinker; and while there are many objectionable speculations and peculiar Eclecticisms—verging, at times, upon the confines of Hœmeopathy—yet, as a materia medica, ingenius and curious, of indigenous remedies—as well as of others thrown in, in absolute defiance of American Eclecticism—it can be read profitably as furnishing hints, suggestions and facts no where else to be found. The work exhibits one phase of medical thought and literature, and as such, deserves a respectful hearing and consideration. We are sure such works have missions for good—good when the wheat is winnowed from the chaff; bad only when used to decry true Eclecticism and to upbuild ingenius but erroneous speculations.

We are indebted to the author for the work, and thank him for the courtesy. We trust he may live long to pursue his investigations, and that in the end he may become an Eclectic indeed.

W. T. G.

An Introduction to Physical Measurements, with Appendices on Absolute Eclectrical Measurements, etc. By Dr. F. Kohlrausch, Prof. in Ordinary at the Grand Ducal Polytechnic School at Darmstadt, etc. Translated from the second German edition. By Thomas Hutchinson Waller, B. A. B. Sc., and Henry Richardson Proctor, F. C. S. New York: D. Appleton & Co. 1874.

This is a very complete work, the larger part being devoted to measurements of physical quantities. To the student devoting himself to the investigation of physical laws it is invaluable, and is vastly superior to mere verbal teaching. W. T. G.

